# Facial pressure zones of an oronasal interface for noninvasive ventilation: a computer model analysis* **

**DOI:** 10.1590/S1806-37132014000600009

**Published:** 2014

**Authors:** Luana Souto Barros, Pedro Talaia, Marta Drummond, Renato Natal-Jorge

**Affiliations:** University of Porto, Faculty of Engineering, Porto, Portugal. Faculdade de Engenharia da Universidade do Porto - FEUP, University of Porto Faculty of Engineering - Porto, Portugal; University of Porto, Faculty of Engineering, Porto, Portugal. Faculdade de Engenharia da Universidade do Porto - FEUP, University of Porto Faculty of Engineering - Porto, Portugal; University of Porto, Faculty of Medicine, Porto, Portugal. Faculdade de Medicina da Universidade do Porto - FEUP, University of Porto Faculty of Medicine - São João Hospital, Porto, Portugal; University of Porto, Faculty of Engineering, Porto, Portugal. Mechanical Engineering Institute, University of Porto Faculty of Engineering, Porto, Portugal

**Keywords:** Noninvasive ventilation, Computer simulation, Respiration, artificial

## Abstract

**OBJECTIVE:**

To study the effects of an oronasal interface (OI) for noninvasive ventilation, using a three-dimensional (3D) computational model with the ability to simulate and evaluate the main pressure zones (PZs) of the OI on the human face.

**METHODS:**

We used a 3D digital model of the human face, based on a pre-established geometric model. The model simulated soft tissues, skull, and nasal cartilage. The geometric model was obtained by 3D laser scanning and post-processed for use in the model created, with the objective of separating the cushion from the frame. A computer simulation was performed to determine the pressure required in order to create the facial PZs. We obtained descriptive graphical images of the PZs and their intensity.

**RESULTS:**

For the graphical analyses of each face-OI model pair and their respective evaluations, we ran 21 simulations. The computer model identified several high-impact PZs in the nasal bridge and paranasal regions. The variation in soft tissue depth had a direct impact on the amount of pressure applied (438-724 cmH_2_O).

**CONCLUSIONS:**

The computer simulation results indicate that, in patients submitted to noninvasive ventilation with an OI, the probability of skin lesion is higher in the nasal bridge and paranasal regions. This methodology could increase the applicability of biomechanical research on noninvasive ventilation interfaces, providing the information needed in order to choose the interface that best minimizes the risk of skin lesion.

## Abstract (pt)

**OBJETIVO:**

Estudar os efeitos de uma interface oronasal (IO) durante a ventilação não invasiva usando um modelo computacional tridimensional (3D) com a capacidade de simular e avaliar os principais pontos de pressão (PP) na face humana.

**MÉTODOS:**

Foi utilizado um modelo digital 3D da face humana, baseado em um geométrico pré-estabelecido. O modelo simulava tecidos moles, crânio e cartilagem nasal. O modelo geométrico foi obtido por varredura a laser 3D e pós-processado para uso no modelo criado. Uma simulação computacional foi realizada para determinar a pressão necessária para criar os PP faciais com o objetivo de separar a almofada da parte rígida da IO. Imagens gráficas descritivas dos PP e de sua intensidade foram obtidas.

**RESULTADOS:**

Para as análises gráficas de cada par de modelos face-IO e suas respectivas avaliações, foram realizadas 21 simulações. O modelo computacional identificou vários PP com alto impacto sobre a ponte nasal e área paranasal. A variação da profundidade nos tecidos moles teve um impacto direto na quantidade da pressão aplicada (438-724 cmH_2_O).

**CONCLUSÕES:**

Os resultados da simulação computacional indicam que, em pacientes submetidos à ventilação não invasiva com uma IO, a probabilidade de ocorrência de lesões cutâneas é maior na ponte nasal e nas áreas paranasais. Esta metodologia pode aumentar a aplicabilidade na investigação biomecânica das interfaces de ventilação não invasiva, fornecendo informações necessárias para a escolha de uma IO que minimize o risco de lesão na pele.

## Introduction

Noninvasive ventilation (NIV) plays an important role in the treatment of acute and chronic respiratory failure.^(^ 1 ^,^ 2 ^)^ Nevertheless, NIV has been shown to fail in 40-60% cases in the acute setting.^(^ 2 ^-^ 4 ^)^ Interface-related problems are one of the most common adverse effects, accounting for 50-100% of all NIV-associated complications.^(^ 4 ^,^ 5 ^)^

The choice of interface is a major determinant of NIV success, mainly because it adversely affects patient comfort.^(^ 6 ^)^ Oronasal masks are preferred for patients with acute respiratory failure, because such patients generally breathe through the mouth to bypass nasal resistance,^(^ 7 ^,^ 8 ^)^ whereas nasal masks are reportedly used in 73% of patients with chronic respiratory failure.^(^ 1 ^,^ 4 ^,^ 8 ^)^ The choice of interface can also play a major role in NIV complications, such as air leak, claustrophobia, facial skin erythema, acne-form rash, skin damage, and eye irritation.^(^ 4 ^,^ 8 ^)^ The most common sites of friction and skin damage are the bridge of the nose and the upper lip (nasal mask); the nasal mucosa (nasal pillow mask); and the axillae (helmet).^(^ 1 ^)^ The creation of a more objective model to aid in the selection of an NIV interface-depending on the setting, the patient circumstances, or even the materials used-is warranted. Promising software and the evolution of computational models over the last decades have made a significant contribution to the development of medical products, creating a link between mechanical engineering and clinical practice. One of the advances in engineering that shows the greatest potential for biomechanical applications is the finite element method (FEM).^(^ 9 ^)^ The FEM was developed by engineers in the 1990s as a means of analyzing the mechanical behavior of complex structures.^(^ 10 ^)^ At present, the FEM is applied in the fields of engineering, science, and medicine. It is a computerized numerical technique that can be used in order to establish the stress and displacement fields in a specific structure. In simpler terms, one can say that the FEM solves complex problems by redefining them as the summation of a series of simpler, interrelated problems. In brief, the FEM subdivides an object into a suitable set of small of discrete regions (the finite elements), which are linked by common points (the nodes). Although the structure under study can be complex and irregularly shaped, the individual elements should be simple and easily analyzed. The elements can be of one-, two-, or three-dimensions and can assume distinct geometries (lines, tetrahedrons, shells, plates, etc.) The behavior of each element is analyzed in terms of the loads and responses at the nodes, and is described by an elemental small matrix, relating a vector nodal displacement to a vector of applied nodal forces. The geometry is so represented in a simplified and discrete way, although still characterizing the object to be modeled.

We hypothesized that the use of a three-dimensional (3D) computational model would increase the likelihood of accurately assessing problematic pressure zones (PZs) in patients submitted to NIV with an oronasal interface (OI), because it would allow the facial anatomy and the corresponding points on the mask to be taken into account. The objective of this study was to evaluate the effects of an OI using a 3D computational model with the ability to simulate and evaluate the main PZs.

## Methods

For this study, we used a digital model of a human face, based on a geometric model described previously.^(^ 11 ^,^ 12 ^)^ The facial geometry was extracted and simplified for our analysis. The 3D model was created with three main parts: soft tissues (part of the scalp, muscle tissue, fat, and skin tissue); skull; and nasal cartilage. The soft tissue part used four-node tetrahedral elements, whereas the skull and nasal cartilage parts used shell elements. We connected the parts using the common nodes in the interfaces. The mandible was free to move relative to the skull, according to the human anatomy (for model performance and discretization of the data, no mouth aperture was modeled). The mechanical properties of the types of formulations used in the model are presented in Table 1. The head was selected along the dorsal plane near the geometrical reference of the head and along the transverse plane below the mandible.

**Table 1 -:** Mechanical properties of the types of formulations used in the model.

Material	Property	Characterization
Soft tissue	Elastoplastic	Johnson-Cook
Cartilage	Linear elastic	Hooke’s Law
Cortical bone	Viscoelastic	Maxwell-Kelvin-Voigt
Mask cushion	Viscoelastic	Maxwell-Kelvin-Voigt

**Figure 1 -:**
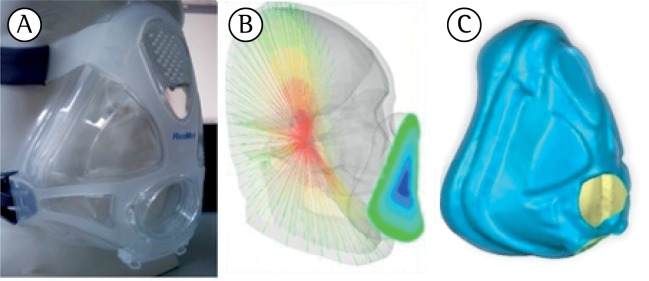
1A: QuattroTM FX mask (no elbow), size L; B: finite element model of A (head and simplified cushion); C: geometric model of A obtained by threedimensional laser digitalization.

**Figure 2 -:**
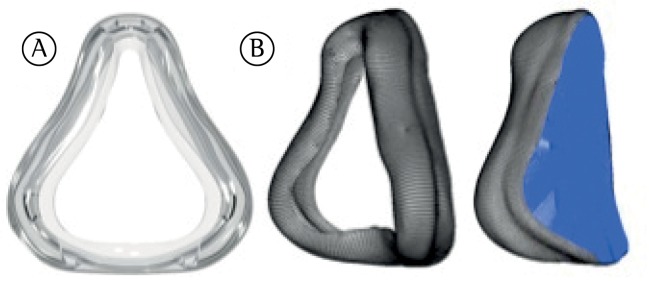
A: QuattroTM FX mask cushion, size L; B: finite elements model of the cushion (A), simplified.

The OI modeled was a simplified version of the Quattro^TM^ FX (ResMed, Bella Vista, Australia). The simplified version was used because it has no internal membrane, which can improve the adjustment of the mask. This geometrical model was obtained by 3D laser scanning and post-processed for subsequent use in the created model (Figure 1). The mask surface obtained was selected with the objective of separating the cushion from the frame. The cushion was then pre-processed (transformed into computer-aided design models that can be manipulated) to create the finite element model (Figure 2). The cushion model is composed of shell elements based on a large deformation formulation.

After the FEM models of the human face and OI had been constructed, we ran a simulation of the interaction between the two in the RADIOSS^(r)^ multidisciplinary finite element solver (Altair Engineering, Troy, MI, USA). At the initial stage, the models are assembled in such a way that there is a gap of approximately 22 mm between the OI model and the human face model, in order to prevent penetration. At the next stage, loads and boundary conditions are applied and the OI is moved toward the human face. Interactions between the two occurred primarily in the frontal region (including the nasal bridge), the maxillary region, and the mandible region. At the third stage (the OI stage), the OI is nearly stable on the human face, with an increasing PZ. The pressure distribution over the contact areas was then determined (Figure 1). As described in the literature,^(^ 11 ^)^ we subsequently applied a pressure load of 25 cmH_2_O to the mask, in order to simulate the tension created by tightening the elastic straps, which is the standard method of attaching OIs during NIV. These elastic strap systems hold the mask to the face while the ventilator pressure is pushing it away from the face, the two operating as opposing forces. From a mechanical point of view, one progressively tightens the strap system until there is no air leakage at peak inspiratory pressures. The pressure was preset at 25 cmH_2_O to insure that it was lower than the skin capillary perfusion pressure. ^(^ 11 ^)^ Descriptive graphical (descriptive analysis) images from the local areas of the PZs and their intensity were obtained. The results presented were recorded for an interval of 40 ms, after the model had stabilized (Video 1; available in the online version of the Brazilian Journal of Pulmonology; http://jornaldepneumologia.com.br/detalhe_video.asp?id=2355/).

## Results

**Figure 3 -:**
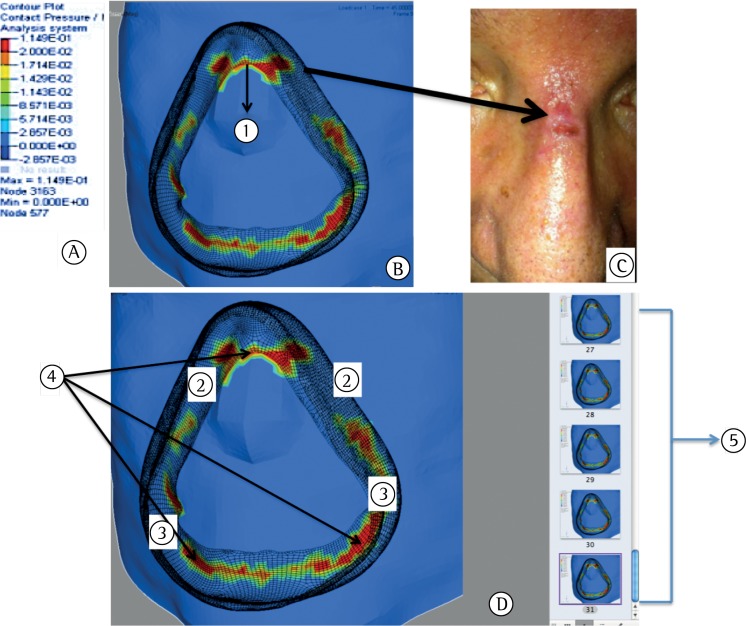
Graphical images of pressure zones (A), in which blue, green, yellow, and red, respectively, indicate ascending values for pressure and contact pressure; pressure zones (B) of an oronasal interface and a corresponding skin lesion (C) induced by one of the high-pressure zones (B1, point of contact between the mask membrane and the nose) of the same interface; in some areas of the mask cushion (D), the contact pressure was absent/low (D2/D3), whereas it was high in other areas (D4), specifically the nasal bridge, paranasal, and mandibular regions. Stages of the simulation, according to the approach and contact with the mask, are shown in D5.

For the graphical analyses of each face-OI model pair and their respective evaluations, we ran 21 simulations. That was the number of simulations deemed necessary in order to adjust the pre-positioning of the mask; characterize the contact; determine how fast the load and pressure should be applied; and ensure that the software would yield a sufficient number of results without errors (the software itself evaluates and demonstrates errors regarding the simulation). Descriptive graphical images of PZs were obtained (Figure 3). The OI presented several PZs with major relevance in the nasal bridge, paranasal, and mandibular regions (Figure 3). There was a significant pressure increase at the point of contact between the mask membrane and the nose (Figure 3). In a graphical analysis of the contact pressure, we observed that the pressure distribution is relatively homogeneous across the entire OI contact area, the contact being broken only at the lateral lip commissure and the frontal zone of the maxilla (Figure 3). In the frontal zone of the maxilla, we observed a large section in which the contact pressure was low or absent. In the nasal bridge and paranasal regions, we observed a PZ associated with an increased density of > 204 cmH_2_O. 

**Figure 4 -:**
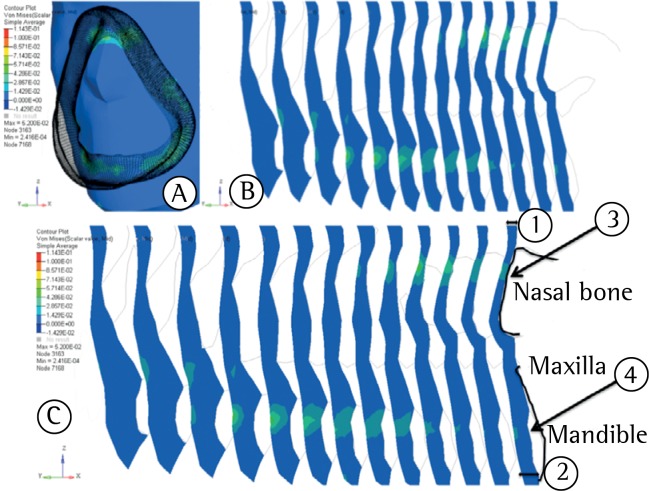
A: Von Mises equivalent stress distribution (a combination of all stress components) on the skin surface; B: pressure variation according to soft tissue depth.

As can be seen in Figure 4, a variation in soft tissue depth translated to a variation in the pressure applied, which ranged from 438 cmH_2_O to 724 cmH_2_O. In a sagittal view, the pressure effects observed that in the upper-lateral zone of the nose were seen to extend to the bone tissue (nasal bone). A similar phenomenon was observed where the cushion aligns with the mandible and maxilla. 

## Discussion

The main findings of the present study were as follows: the likelihood of a PZ was highest in the nasal bridge and paranasal regions; there was a significant pressure increase at the point of contact between the mask membrane and the nose; and the variation in soft tissue depth had a direct impact on the amount of pressure applied. Our findings are in accordance with the literature in terms of the area where skin lesions typically occur (the nasal bridge).^(^ 5 ^,^ 12 ^)^ Nasal bridge ulceration, as depicted in Figure 3, is a relatively common complication of NIV, occurring in up to 10% of ventilated patients.^(^ 5 ^)^

Skin lesion at the site of mask contact is the most common complication of NIV,^(^ 4 ^)^ and skin necrosis is normally due to excessive mask-fit pressure, which prevents adequate tissue perfusion.^(^ 11 ^)^ Schettino et al.^(^ 11 ^)^ described a simple method of ensuring appropriate mask attachment during NIV, which is to measure the mask-fit pressure within the pneumatic cushion of the mask. To our knowledge, ours is the first study accessing the behavior of an OI on PZs and contact pressure during NIV using the FEM techniques. The PZ at the point of contact between the mask and nose is often evaluated only as incorrect use of the OI. Our preliminary results indicate PZs in the nasal bridge and paranasal regions, as well as disproportionate contact pressure between the mask membrane and the nose. However, further studies are needed in order to clarify this issue.

On the basis of these preliminary results, we can suggest that the likelihood of skin lesion is greater in skin regions where the proportion of soft tissue is lower. In the human anatomy, the soft tissue is composed of skin, subcutaneous fat, and muscle tissue. The thickness of the soft tissue would be responsible for a variation in the resistance to pressure applied to the skin. In other words, soft tissue of greater depth did not allow pressure to propagate to the bone tissue (of the mandibular or maxillary bones, in the present study). It is possible that, concerning soft tissue depth, a broader distribution of the pressure reduces the likelihood of injury. Low contact pressure of the OI with the right and left lip commissures, as well as with the frontal zone of the maxilla, might be related to increased air leakage, which would corroborate data found in the literature.^(^ 4 ^)^

The development of powerful computational tools provides new approaches to the study ventilator adaptation issues. For example, Lei et al.^(^ 13 ^)^ created digital representations of the complex geometries of a human head and a respirator face-piece, using laser scanners. The authors then manipulated the 3D images and submitted them to a computational analysis of the interface between the respirator and the human face to calculate seal pressure distributions. Some researchers have focused on the sealing pressure distribution between an OI and the human face for an N95 respirator (protective mask) and for a jet pilot oxygen mask.^(^ 13 ^,^ 14 ^)^ The first author to use the FEM to calculate the pressure between a jet pilot oxygen mask (MBU-20/P) and the human face was Bitterman.^(^ 15 ^)^ According to Yang et al.,^(^ 16 ^)^ the maximum respirator pressure is 3,344 cmH_2_O, higher than that found in our study. Piccione & Moyer^(^ 17 ^)^ developed a mask "fit and discomfort model" to evaluate fit, protection, and discomfort according to contact location, pressure, shear, and friction. Cohen^(^ 18 ^)^ described an experimental method for evaluating mask seals by measuring seal pressure distributions. We believe that this 3D computational simulation method could predict the PZs between the human face and an OI designed for NIV. Further studies are certainly needed in order to validate and expand this methodology, which shows real promise for applications in mask design and testing.

The present study has a number of limitations. The model of the face was not perfectly analogous to the human anatomy, in which the soft tissue is composed of skin, subcutaneous fat, and muscle tissue. In addition, the pressure was applied over a single point, rather than being distributed across the mask, as it would be in a real-life setting, and we did not adjust the model to account for the effects of strap tension. Furthermore, we did not take the time factor into account. Moreover, the OI employed (a simplified model) has only one internal membrane. Theoretically, the mask membrane can change the pressure values and the distribution of that pressure over the face. Therefore, our findings cannot be extrapolated to the commercialized version of the mask.

These preliminary results support the idea that the probability of skin breakdown is highest in the nasal bridge and paranasal regions in patients on NIV with an OI. This methodology could introduce the biomechanical study of NIV interfaces as a strategy to minimize lesion. There is a need for quantitative validation of this model, including the internal membrane of nasal pillow masks.
